# Comparison of three different fixation constructs for radial neck fractures: a biomechanical study

**DOI:** 10.1186/s13018-017-0680-2

**Published:** 2017-11-14

**Authors:** Hongwei Chen, Dengying Wu, Tianlong Pan, Jun Pan, Rui Zhang, Xuchao Shi

**Affiliations:** 1 Department of Orthopaedics Surgery, Yiwu People’s Hospital, NO.699, Jiangdong Road, Yiwu, Zhejiang Province 322007 China; 2 Department of Orthopaedics Surgery, The Second Affiliated Hospital and Yuying Children’s Hospital of Wenzhou Medical University, NO.109, Xue Yuan West Road, Wenzhou, Zhejiang Province 325027 China

**Keywords:** Radial neck fractures, Screw, Biomechanical comparison, Different fixation constructs

## Abstract

**Background:**

Fixation of radial neck fractures can be achieved with a plate and screw construct or with two screws. This study evaluated the biomechanical properties of three different fixation methods following radial neck fractures.

**Methods:**

Twenty-four fourth-generation composite radii were sawed to simulate an unstable radial neck fracture. They were then instrumented with a plate and screw construct or two different orientations (crossed and parallel) of screw fixation. Implants were tested under bending and torsional loads via a tension torsion composite test system. Bending and torsional failure loads were added to the remaining implant-radius constructs if they did not fail during the previous tests.

**Results:**

During the bending loading test, the crossed-screw group showed the greatest stiffness, followed by the parallel-screw group, the plate group demonstrating the weakest stiffness. There was no significant difference between the crossed- and the parallel-screw groups. However, there was a significant difference between the two screw groups and the plate group. During the bending failure test, the largest stiffness was found for the crossed-screw group, while the plate group exhibited the smallest stiffness. There was a significant difference between the three groups. During the torsion loading test, the highest stiffness was observed for the crossed-screw group, while the plate group showed the lowest stiffness. In the torsion failure test, the failure torques were 11.97 ± 2.659, 8.531 ± 1.768, and 7.079 ± 1.666 N m respectively for the crossed-screw, parallel-screw, and plate groups. There was a significant difference between the crossed-screw group and the two other groups.

**Conclusions:**

Crossed screws and plate fixation are commonly used in clinical practice to treat simple radial neck fractures. While the present study shows that the parallel-screw method results in similar biomechanical strength as the two other techniques, it has the advantages of reaching limited wound exposure and having the implant buried. Therefore, it may be widely used in clinical practice.

## Background

Radial head and neck fractures are uncommon, their reported incidence being approximately 55.4 per 100,000 persons [1]. The injury mechanism of the radial neck is usually an axial load caused by valgus and fall [2, 3], during which the radial capitellar joint usually transfers 60% of the upper limb load [4]. In order to restore stability and alignment of the displaced radial head and neck following a fracture and to enable then an early range of motion, open reduction with internal fixation (ORIF) is essential [5–7]. There are controversial and varied treatment choices for radial head and neck fractures. However, there is still no consensus regarding the best treatment to dispense for Mason–Johnston types II–IV fractures [8]. The Mason classification has been widely used to describe the radial head and neck fracture [9]. Broberg and Morrey [10] modified this classification with type II fractures being those having more than 2 mm of displacement and involving at least 30% of the radial head. Johnston [11] then added a type IV fracture to the classification, which corresponds to a radial head or neck fracture associated with an elbow dislocation. The purpose of this study was to determine the biomechanical properties of the bending and torsional stiffnesses of a plate and two different screw fixation orientations (crossed and parallel) in an unstable radial neck fracture. Only five studies evaluating the biomechanical characteristics of various radial head implants were found in the literature [12–16].

A simple radial neck fracture model was used to standardize our investigation. Although this model does not reproduce radial head and neck fractures, the results of this study can still help orthopedic surgeons to develop the most reasonable internal fixation pattern. We aimed at comparing the stiffness and strength of the plate and two different screw orientations, the plate and the crossed screw being commonly used in clinical practice, and the parallel screw being specifically designed by us for this study.

## Methods

We used 24 identical (i.e., same size and density) synbone radii (SYNBONE AG, Malans, Switzerland). Each radius was cut at the mid-shaft level, leaving an approximately 10-cm long proximal segment. A transverse osteotomy was then made at the head-neck junction by using a micro-sagittal saw, this simulating a longitudinally unstable radial neck fracture.

**Fig. 1:**
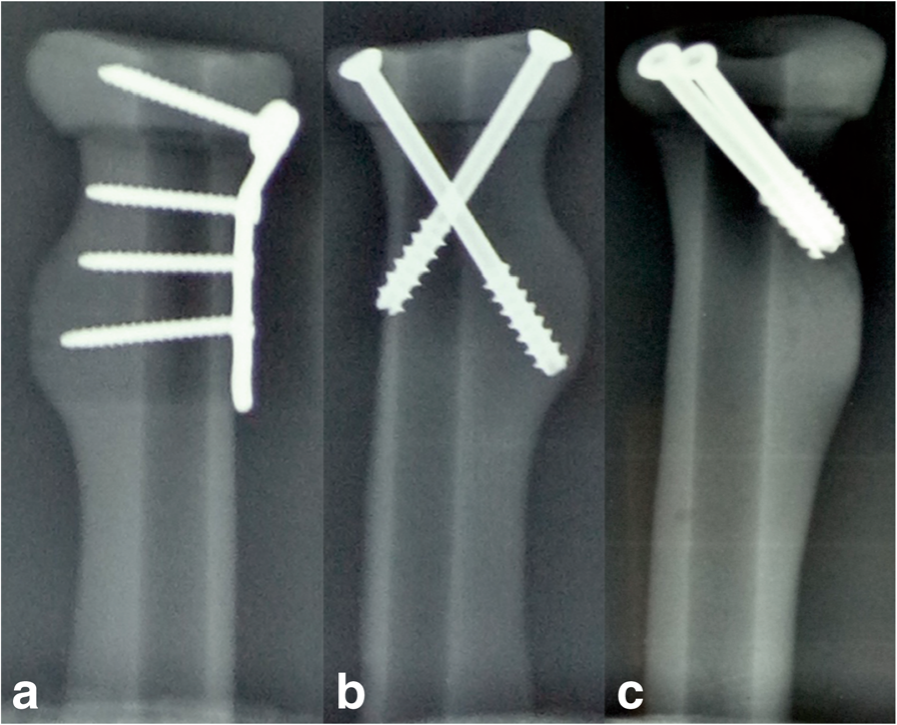
Radiographs of the plate (**a**), crossed screw (**b**), and parallel screw (**c**)

**Fig. 2:**
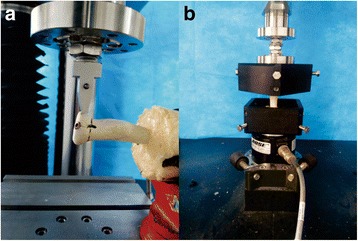
The radial head model was placed in the instrument for bending (**a**) and torsional (**b**) loading

Three different fixation devices were tested for reconstruction after the osteotomy: a radial head plate and screws (Stryker, Mahwah, NJ, USA) or two different orientations (crossed and parallel) of screw fixation (AO, Davos, Switzerland). The plate group included a plate construct involving five bicortical screws. In the crossed-screw group, the screws were placed approximately 60° apart, as described by Smith and Hotchkiss [17]. In the parallel-screw group, the screws were inserted in parallel to each other. The two screws were inserted into the radial head from the outer edge of the top at 45° of the radial head axis. The length of the two screws was uniform, and the distance between the two screws was 5 mm. The plate was placed in the safe zone of the radial head, which lies on the dorsal surface of the radius [18]. The fixations were evaluated using X-ray images. Figure 1 shows X-rays of the reconstructed radial heads with the three different fixation devices described above. The transversely cut end of the radial shaft was then potted in a metal tube by using polymethylmethacrylate (PMMA). Figure 2 displays some representative potted specimens. An Electro Force 3510 Tension torsion composite test system (Bose, MA, USA) was used to test the specimens. The testing machine features up to ± 75,000 N of axial force capacity and ± 50 N m of torque capacity.

### Bending load test

The bending load was applied to the radial head through a custom solid cup made of PMMA. The loading orientation was posterior to anterior. Before the actual test, a preload of 10 N was applied three times at the same velocity (2 mm/min) to the radial head which had to resist to a horizontal slide. This position was regarded as the baseline to record the displacement of the head and the data were then cleared. Next, the construct was loaded in compression at a rate of 2 mm/min. The test was stopped when the displacement of the radial head reached 2 mm.

After the bending load test, if the fracture models did not fail, a failure load test was performed at a rate of 2 mm/min. Bending stiffness and bending failure loads were recorded.

### Torsional load test

First, the head of the radial was coupled with the actuator of the Electro Force 3510 Tension torsion composite test system with the use of an additional double gimbal fixture (Bose, MA, USA). Radii were then loaded for 10 cycles at 1 Hz in both the anterior and posterior torsional direction at five levels: − 0.5°, − 1.0°, − 1.5°, − 2.0°, and − 2.5°. The torsional stiffness obtained was then used to evaluate the ability of the fixed structure to resist rotation.

Similarly to the bending load test procedure presented above, the fracture models underwent a failure load test at a rate of 5°/min if they did not fail after the torsional load test. Torsional stiffness and torsional failure loads were recorded.

Failure of the model was defined as (1) a new fracture line appearing in the model in addition to the original fracture line; (2) an internal fixation failure, such as plate or screw bending, cutting, or fracture; (3) a lateral displacement of the radial head superior to 5 mm or a torsion displacement of the radial head that exceeded 14.5°; and (4) a flat load-displacement curve in the data acquisition image or an absence of change in the displacement of the model while the load still increased.

The stiffness was defined as the slope of the regression line fitted to the loading segment of the cyclic load displacement curves. Data of each group are presented as mean ± standard deviation (SD). For statistical analysis, SPSS 21.0 (IBM Corporation, Armonk, NY, USA) was used. Mechanical parameters were compared by using one-way analyses of variance. The level of statistical significance was set at *p* < 0.05.

## Results

**Fig. 3:**
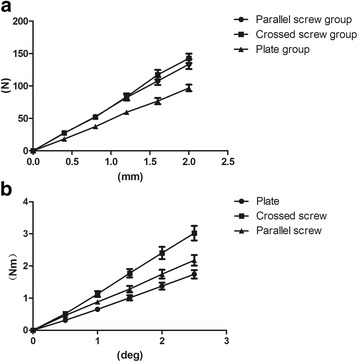
Comparison between the plate group, crossed-screw group, and parallel-screw group. The slopes of the curves reflect the bending stiffness in **a** and torsional stiffness in **b**

**Fig. 4:**
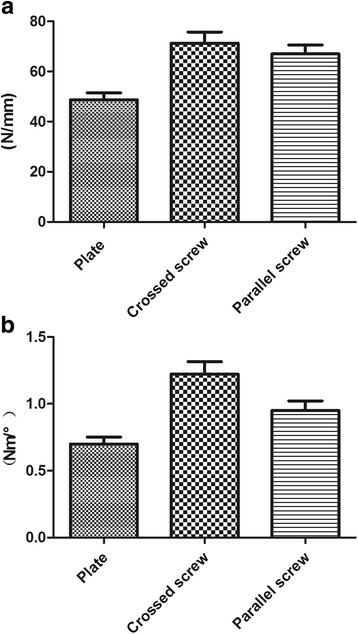
Comparison of average bending (**a**) and torsional (**b**) stiffness of the plate group, crossed-screw group, and parallel-screw group. Standard deviation is represented with the range bars on top of each graph

**Fig. 5:**
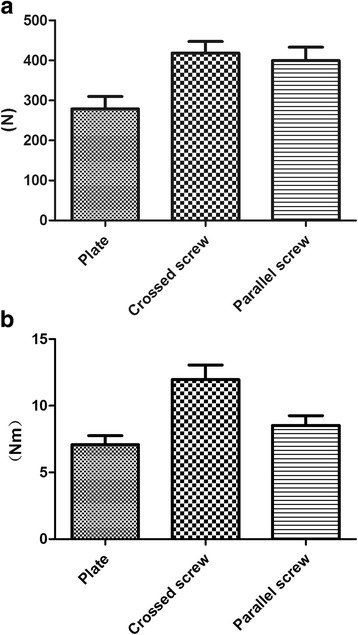
Comparison of bending (**a**) and torsional (**b**) failure loads of the plate group, crossed-screw group, and parallel-screw group. Standard deviation is represented with the range bars on top of each graph

We compared the stiffness of the three structures from the five bending load levels (Table 1). The stiffness of the crossed-screw group was the largest compared with the two other structures. Although the stiffness of the parallel-screw group appeared smaller than the crossed-screw group, our results revealed no statistical difference for all levels (Table 2). The stiffness of the plate group was smaller than that of the parallel-screw (*p* = 0.003) and crossed-screw groups (*p* < 0.001). All bending load data were processed as a displacement-load curve, as depicted in Fig. 3. The three curves in the figure represent the three different load-displacement variations. It can be observed that the load-displacement variation was approximately linear in the range of 0–2 mm for the three groups. A first analysis revealed that the three sets of data met the homogeneity of variance criterion. The average stiffness of the plate group was 48.73 ± 6.801 N/mm. The parallel-screw group was 25.28% stiffer than the plate group while the crossed-screw group was 46.21% stiffer than the plate group (Table 3). A stiffness comparison between the three groups is presented in Fig. 4. The bending failure load test revealed that the failure load was the largest for the crossed-screw group (418.51 ± 70.68 N), whereas the minimum failure load was observed for the plate group with only 279.22 ± 75.36 N, the parallel-screw group standing between the two groups with 399.73 ± 81.60 N (Fig. 5). There was no statistical difference between the two screw groups. However, the plate group was statistically different from the two other groups.

**Table 1:** Bending stiffness of plate, crossed-screw, and parallel-screw constructs

Load (mm)	Mean ± SD (N/mm)		
	Plate group	Crossed-screw group	Parallel-screw group
0.4	45.56 ± 7.23	68.86 ± 10.07	68.24 ± 19.82
0.8	46.69 ± 5.31	65.11 ± 10.60	66.01 ± 10.61
1.2	49.68 ± 6.98	69.53 ± 10.46	68.57 ± 9.87
1.6	47.98 ± 7.46	73.37 ± 11.16	67.29 ± 9.26
2.0	48.44 ± 6.29	69.66 ± 10.65	66.82 ± 9.30

**Table 2:** Comparison of three constructs during bending

Constructs’ type (average N)	*P*		
	Plate group	Crossed-screw group	Parallel-screw group
Plate group		0.000	0.003
Crossed-screw group	0.000		0.427
Parallel-screw group	0.003	0.427

**Table 3:** Average stiffness on bending and torsion of plate, crossed-screw, and parallel screw constructs

	Plate group	Crossed-screw group	Parallel-screw group
Bending average (N/mm)	48.73 ± 6.80	71.25 ± 10.88	67.05 ± 8.54
Torsion average (Nm/°)	0.69 ± 0.12	1.22 ± 0.22	0.95 ± 0.17

We also compared the behavior of the three structures at the five different levels during the torsion loading test (Table 4 and Fig. 3). Average torsional stiffness is presented in Table 3 and Fig. 4 for the three structures. The crossed-screw group demonstrated the greatest stiffness, followed by the parallel-screw group and then the plate group. As presented in Table 5, statistical results revealed that the average stiffnesses of the three groups were significantly different from each other (*p* < 0.05). Results of the torsion failure test showed that stiffness was significantly higher for the crossed-screw group than for the parallel-screw and plate groups, with no significant difference between the parallel-screw group and the plate group (Fig. 5).

**Table 4:** Rotation stiffness of plate, crossed-screw, and parallel-screw constructs

Rotation degree (°)	Mean ± SD (Nm/°)		
	Plate group	Crossed-screw group	Parallel-screw group
0.5	0.31 ± 0.08	0.51 ± 0.13	0.46 ± 0.09
1.0	0.65 ± 0.09	1.12 ± 0.22	0.88 ± 0.18
1.5	1.01 ± 0.19	1.77 ± 0.31	1.28 ± 0.24
2.0	1.37 ± 0.26	2.4 ± 0.46	1.74 ± 0.33
2.5	1.74 ± 0.31	3.02 ± 0.55	2.17 ± 0.40

**Table 5:** Comparison of three constructs during torsion

Constructs’ type (average Nm)	*P*		
	Plate group	Crossed-screw group	Parallel-screw group
Plate group		0.000	0.029
Crossed-screw group	0.000		0.019
Parallel-screw group	0.029	0.019	

## Discussion

The surgical management of radial head and neck fractures has evolved over the last decades. For radial head and neck fractures of type II, there is no general agreement in the literature on the superiority of any surgical or conservative treatment over the other. Radial head fractures of type III are commonly treated with surgery. Several surgical treatment options can be performed: ORIF by screws, plates, k-wires or biodegradable pins, implantation of a prosthesis [19, 20], or resection of the radial neck [21–23]. Regarding the initial stability of the forearm and elbow, and the later development of arthritis, ORIF is believed to be a superior technique compared with the radial head resection for the treatment of unstable, displaced radial head fractures [5, 24, 25]. Ikeda et al. [25] compared the clinical results obtained after either resection or ORIF of Mason type III fractures. They reported a better outcome with better function for patients in whom the radial head was reconstructed than for patients whose radial head was resected.

Most studies have reported ORIF results for fractures of both the radial head and neck. However, only few studies have published results on adult-only radial neck fractures. Esser et al. [5] followed up for 7 years 26 patients who were treated with ORIF via a plate fixation. None of them presented with bad outcomes. Nevertheless, many articles pointed out that the treatment of radial neck fracture with plate fixation may produce a variety of complications. In 2007, Smith et al. [16] reported that 6 out of 10 patients were not satisfied after being treated with a plate. In a study by Li et al. [26], 58 patients were reviewed for 1 year. The mean range of forearm rotation in the screw group was significantly better than that in the plate group, and the screw group had a lower incidence of heterotopic ossification than the plate group. Based on these studies, we asked ourselves whether screw fixation was a better technique than using a plate to treat radial head and neck fractures. A simple biomechanical study of the fracture of the radial neck was made by Gutowski et al. [15]. They compared two oblique headless compression screws and a radial neck plate. They concluded that the two strategies provide similar strength and stiffness for the fixation of transverse radial neck fractures. However, the two oblique screws might be preferred for simple transverse neck fractures since this strategy requires limited wound exposure and the implant is buried. The above two studies seem to indicate that the use of screws is better than the use of plate for treating radial neck fractures.

In our study, the stiffness of the radial neck fractures was compared between the three fixation methods in order to evaluate the effect of these structures on fracture stability. These structures can enable the injured patients to perform postoperative functional exercise earlier. Since bending and torsion are the main forces applied to the radial head and neck during normal elbow movements, we used these two force types as our loading parameters. During the bending test, the crossed-screw group and the parallel-screw group had similar stiffness whereas the smallest stiffness was observed for the plate group. We believe that the screw groups were directly connected through the internal ends of the fracture. In the plate group, the plate was fixed with screws in one end of the fracture, and there was no rigid connection within the fracture site. Also, only the lateral side of the fracture end was connected by the plate, and the fixed force was in the lateral fracture side. The load of the crossed-screw group was larger than that of the plate group in the bending failure load test, which is consistent with the results reported in Gutowski’s study.

The stiffness of the crossed-screw group was the largest in the torsion test, whereas the stiffness of the plate group was the smallest. This suggests that the crossed screws have a good anti-torsion effect, can provide good fracture stability, and promote early functional exercise without any displacement of the fracture ends. During the failure test, the crossed-screw group shows a significantly larger stiffness than the parallel-screw group and the plate group. We believe that at the proximal end of the fracture, the fixation of the parallel screws and the plate were eccentric, whereas it was in the center distribution of the radial axis for the crossed-screw group. During the torsion process, only one side was forced on the fracture ends of the plate and parallel-screw groups, while the two sides of the fracture were stressed in the crossed-screw group. Therefore, the stiffness of the crossed-screw group was larger than the two other groups.

**Fig. 6:**
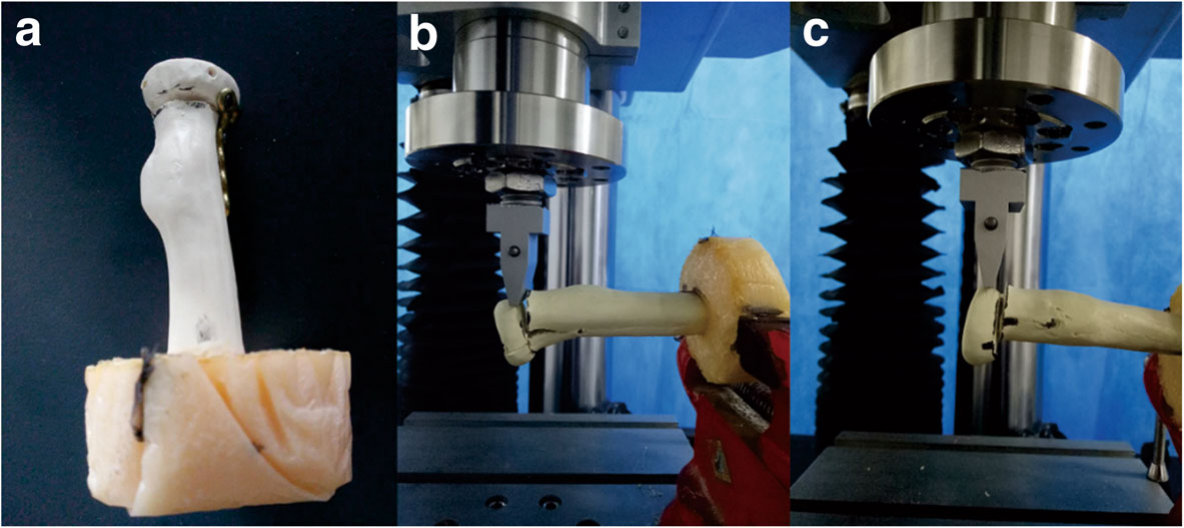
Different failure mode of the plate (a), crossed screw (b), and parallel screw (c)

As shown in Fig. 6, the failure modes were different among the three groups. In the plate group, the plate was deformed at the fracture site, but the screws on the plate did not shift. In the parallel-screw group, a new fracture was noticed in the proximal part of the fracture, but no change was observed in the distal part of the fracture. In the crossed-screw group, one or two screws at the distal end of the fracture were cut out.

Although the parallel screw has not been used in clinical practice yet, the biomechanical results for this fixation method are considerable. In clinical practice, the wound exposure of the screw treatment of the radial head and neck fracture is less than for the plate fixation method. While the crossed screws need to be taken from both sides of the radial head and although the wound exposure is small, the two entry points cannot simultaneously be exposed without rotating the forearm of the fixed operation. Moreover, this may lead to complications when the two screws collide with each other, or when the two screws are not in the center of the radius occurred (i.e., occurrence of an offset). Also, the parallel-screw method only requires a small wound exposure, the two screws being inserted in parallel at a 45° angle with the radial axis in order to fix the fracture site. The biomechanical results obtained in the parallel-screw group are just slightly less than those obtained for the crossed-screw group. Therefore, we can consider using two parallel screws to fix a simple radial neck fracture.

Although our study provides interesting results regarding the biomechanical properties of three different fixation methods, it also has some limitations. First, since the biomechanical properties of the standard bone were investigated without any muscle and other corresponding soft tissue attachment, it cannot simulate the real human elbow joint force transmission and role. Second, our sample size was relatively small. Bending load and torsion direction of the body cannot completely simulate the real daily activities of the human body or the mechanical injury mechanism. In addition, it should be noted that there is a subtle difference in screw orientation in the coronal plane. We attempted to control for this by predrilling the screw trajectory with a custom-made jig, but our funds were limited, and we could not proceed this way. Also, the biomechanical analysis provided in this study only reports bending, torsional, and failure loads. The observed index only includes bending and torsional stiffness as a trade-off. Other biomechanical performance indicators are lacking. Finally, the use of synthetic bone models as opposed to cadaveric specimens could also be seen as a limitation of this study.

## Conclusion

Results of this biomechanical study suggest that the crossed-screw fixation is optimal for Mason II radial neck fractures among the three internal fixation strategies analyzed in this study (crossed screws, parallel screws, and plate). Alternatively, the parallel-screw method also constitutes a good internal fixation strategy. The stiffness of the plate was the weakest among the three investigated techniques and was also the one that required the largest wound exposure. However, our conclusion needs to be supported by additional large sample size studies investigating its biomechanical and clinical application.

## Abbreviations

ORIF: Open reduction with internal fixation
PMMA: Polymethylmethacrylate
